# Southern Dental Association

**Published:** 1886-10

**Authors:** 


AMERICAN JOURNAL

OF

DENTAL SCIENCE.

VOL. XX. THIRD SERIES—OCTOBER, 1886. No. 6.

ARTICLE I.

### SOUTHERN DENTAL ASSOCIATION.

EIGHTEENTH ANNUAL MEETING.

AT NASHVILLE, TENN., JULY 27, 28, 29 AND 30.

(Reported by Mrs. M. W. J.)

[Continued from September Number.]

One after another of the sciences has been brought in till we are liable to have all the *isms* and all the *pathies* which destroy human life rather than preserve it.

The damage which has been done abroad we are not responsible for; the profession at large has nothing to do with those illegal diplomas. They have been sent abroad by disreputable institutions, but we are not responsible for that.

G. F. S. Wright, Columbia, S. C.: Will Dr. Morgan please tell us something about the honorary degree; how it is obtained?

Dr. Morgan: It is bestowed only upon men who are highly recommended; who have made a good showing; men who have made valuable contributions to literature or science. It is only bestowed where well merited. and can do no harm to the profession.

On motion of Dr. McKellops, the subject of Dental Education was temporarily passed, the hour of adjournment having arrived.

The hour from 12 to 1 o'clock was, on motion of Dr. McKellops, set apart for the exhibition and examination of appliances, etc.

On motion, adjourned to 9 A. M.

---

WEDNESDAY, July 28.

SECOND DAY.—MORNING SESSION.

Called to order at 9 A. M., the President in the chair.

Adjourned to Amusement Hall, on Broad street, seeking cooler quarters and less echo.

Called to order at 10:15 A. M.

Minutes of preceding meeting read and approved.

DENTAL EDUCATION CALLED.

Dr. G. W. McIlhaney reported no other papers.

Dr. Morgan introduced Dr. Hubbard, of Mehary College, an institution for the higher education of colored people.

Dr. Hubbard said that he was glad to have the opportunity of speaking a few words relative to an institution of which comparatively little was yet known by the people generally. It affords opportunities, such as have never before been offered to the colored race. The medical department has been in operation for several years, and sixty-two graduates have gone forth, and are now practicing medicine in the Southern and South-western States. They have been most kindly received by the medical profession, and assistance given in locating.

The medical course covers three years, with daily recitations in physiology and chemistry; two hours daily for ten weeks in the clinical laboratory, etc. The examinations are rigid, 75 per cent. being the minimum. Those who fail to attain this point must re-enter for another year before they can receive the degree of M. D. A dental department is about to be opened in close connection with the medical department, with special instructions in dentistry proper, having a dental laboratory and infirmary. It is designated to do thorough educational work in this department, and the dental profession was asked to extend to graduates the same kind assistance and encouragement as have been given by the medical profession. The work ot the dental department will be extremely philanthropic, the charges being only $30.00 per session. From $75.00 to $80.00 will cover the whole course, including materials, fees, etc. The only requirements for entrance is a good moral character and a fair preliminary education. If students cannot pass the examination for the latter, they are entered for one year in the literary department.

On motion, the subject of Dental Education was passed.

### DENTAL HYGIENE.

Dr. B. H. Teague, Aiken, S. C., read a paper on

### THE PERSONAL HYGIENE OF THE DENTIST.

Dr. Woodley's paper was presented later.

No other papers being offered, the subject was opened for discussion.

Dr. G. H. Winkler, Augusta, Ga., said that he had listened to the paper with interest. He had found the best motor for the engine to be "a big strapping negro." He had tried the power-fan, but after using it for two years, he suffered for two years more from rheumatism in the right shoulder and back of the neck. The constant breeze was delightful for both patient and operator at the time, but his experience had proven to him that it was not safe.

Dr. Teague thought that Dr. Winkler's motive power would be found *too strong* in hot weather.

Dr. Winkler replied that alkali neutralizes cupric acid!

Dr. W. H. Richards, Knoxville, objected to the three meals per day, with dinner at noon, recommended in the paper. Dental operations after a full meal at noon, were not conducive to digestion. He would prefer to work on through the middle of the day, when there was a good clear light, and have a leisure dinner at six after the day's work was done.

Dr. Adair preferred a Southeastern light to the Northern light recommended in the paper; he considered that a better light and more conducive to health; as a rule dentists live too much in the shade; they should have plenty of sunshine properly regulated by blinds.

Dr. J. Hall Moore, Richmond, Va., had tried the Southeastern light, but found that his eyes suffered from it, and that it made his work harder. Preferred the Backus water motor; with it can get into every position—either side of, behind, or in front of the patient; he believed it had added ten years to his life; he would recommend it to every operator; the motor is fixed, and the engine is fixed, but the arm has the greatest freedom of range. The turbine motor is probably more powerful, with less consumption of water.

Dr. W. H. Morgan, thought the profession, as a rule, did not give sufficient attention to these little things. For fifteen years he had offered a prize of $100.00 for the best paper on this subject; a large number of papers had been handed in, but they were all on general hygiene—not on the special hygiene of the dentist. One statement in the paper contained great sophistry—that was the value of light suppers—that is contrary to the rule of all nature; the infant sleeps soundly only when its appetite is satiated, and it is the same through the whole range of the animal creation, they fill the stomach and then rest. Man should not contradict nature. If a hearty supper disagrees it is because of long cultivated habit; each individual should exercise prudence in diet, and be governed by what he finds suits himself best. As to light, the best light in the world is a sky light—light coming from over head; the brow shades the eyes and protects them; a light from the rear is the worst in the world. All light from behind and the side should be shut off; having the light full in the face of the patient, but not in the face of the operator. The best motor is a young intelligent assistant, a young woman decidedly preferable. Perpetual motion was not what was wanted for an engine; an intelligent assistant is an absolute neces sity for a man in full practice—wanted for the engine, the mallet, the fan, to select instruments, etc—a lady is easily trained to understand the slightest motion of the head—no words are necessary; a lady is preferable because she looks at the right end of the instrument; a student looks at the point and not at the handle, and is thinking about what you are doing, not about what he has to do himself. A lady assistant will also receive and entertain patients and wait on them as a man cannot do, attending to their comfort and convenience, and keep the office neat and attractive.

Dr. E. S. Chisholm, Tuscaloosa, said that the subject of hygiene was of the greatest importance to the dentist. He should look carefully to his own comfort and ease of position, avoiding all strain and nervousness. He should occupy rooms having a southern exposure, and plenty of sunlight; light from overhead, protected by an awning was the best. In the Southern States the cool breezes come from the south and southwest, making rooms on the south side cooler in summer, though warmer in winter; a breeze through the rooms carries away all odors from the patient or from the spittoon, or of remedies used. Rooms on the second floor are preferable also—are less subject to malaria. A small light stool which can be readily moved about, allowing the operator to have a sitting position, or alternate sitting or standing, is a prime necessity. Fresh air is all important, but a draft of air on the back is injurious. He preferred a young female assistant, as being handy, neat, cleanly, looking at the right end of the instruments, and looking to all little points of comfort and convenience of patients. Men were clumsy, rough and bungling about such things.

Dr. H. J. McKellops, St. Louis, said he had in his hand a little book entitled "Disinfectants and their Use," which was quite apropos to the subject. Enough attention had not been given to the matter of healthy rooms, sunlight, fresh air. He wished to direct attention to the little book mentioned. Peroxide of hydrogen and menthol as a mouth wash would keep the mouth clean and pure, and would remove the green stains found on the teeth of young chiidren; is a valuable disinfecting agent used as a spray, free from unpleasant odor, and harmless. Chlorine is a more active germicide than bi-chloride of mercury, and would be found to add much to home and office comfort; it has the power of decomposing and destroying the deadly poison of the mad-dog, being a proplylactic agent against the most horrid disaster possible to man, being a *preventive* which is better than a curative agent. He wished the article on chloride would be cut out and posted in every parlor; with all our surroundings pure, sweet and healthy, we should banish disease and suffering. He always had a lady assistant, and he saw to it that she was as well taken care of as she took care of his instruments and other belongings, and every one he owned was cleaned every day; he never had to take up an instrument and look to see if it was clean.

Dr. Rembert, Natchez, said the main points of an office were light, ventilation, temperature and disinfectants. He preferred a north light. He had a male assistant for his motive power, who was also trained to select his instruments as needed; he does not use the mallet. He now uses an operating stool, but had suffered tortures unexpressible before he learned to operate sitting down. Dentists suffer too much worry, from not systematizing their appointments, having perhaps a dozen patients waiting at once. After a day of worry there is no appetite for dinner. Six hours a day is enough except for extracting teeth. Exercise is absolutely indispensable, but it should not be excessive. An early morning ride, or a stroll is the best preparation for the day's work. He did not agree with the paper on the subject of suppers; sleeplessness is often caused by want of food; if a man would go and eat something when restless and sleepless he would probably sleep like a child. The evening should be spent in social enjoyment.

Dr. G. Chisholm, Columbus, Miss., said it was difficult for the dentist to regulate things as he would like to have them. No laws could be laid down for the regulation of all offices alike; each must be guided by his own circumstances. He believed in a free circulation of out-door air; a dozen fans with doors closed would be injurious instead of a benefit. As to excluding night air, he did not see what else we could have at night. In malarial regions out-door air was as necessary as anywhere else. For spittons, etc., he had never seen anything he liked so well as hydro-napthal. The spitton should be cleaned and disinfected after every patient, and never left a whole day. For motive power he used a young man, who was well trained as to the use of instruments, whose duty it was to select them as wanted, to clean them, and to put them back, selecting what was wanted for each patient. He preferred a southern exposure, and thought it only necessary for one to try it a little while to be convinced of its superiority. A skylight was objectionable from the varying clouds.

Adjourned at 12 M., for the EXHIBITION OF APPLIANCES.

Dr. H. J. McKellops exhibited and explained the instruments and appliances for the Herbst method, which he had recently obtained from Dr. Herbst, whose clinics he had attended. There were agate points for filling—German silver for making matrices of which he solders the ends together around the tooth, burring open one side for removal. He cuts the point off a common pin and sticks it between the teeth to hold the dam in place. He rotates the first layer of gold in with hand power, using every form of gold, even to 60; he burrs the gold round against the walls of the cavity, and burnishes with smooth round agate points; the burring makes the gold cohesive, though he sometimes anneals it. Dr. Herbst makes his barbed broaches of 14 caret gold wire; if it breaks off it can be driven up for filling, and no sweating for hours getting out a broken broach. Amen to steel broaches! A special feature of Dr. Herbst is his utilization of all the little odds and ends of everything, displaying wonderful versitility and ingenuity; anything that he picks up is made to answer his purpose, and nothing is thrown away.

A box of specimens sent by Dr. Dunlap was exhibited, including some jaw-bones from ancient Indian mounds. Dr. J. J. R. Patrick pointed out the fact that they showed very plainly the marks of alveolar abscess, and other diseases, supposed to be due to modern civilization. In one there was the remains of a decayed tooth; in another the bony cicatrix of an alveolar fistula. The American Indian suffered from his teeth the same as the white man of to-day. In a hundred skulls it is difficult to find a perfect set of teeth.

Dr. Staples, Sherman, Texas, showed a foil folder, and crumpler or crimper; also a paper-disk carrier. Dr. H. E. Parr has separators, which are quite different from Dr. Perry's but very effective.

Dr. Morgan called attention to a book called "Dental Questions and Answers"—a valuable little *vade mecum* to aid students in reviewing lectures, being a summary of the Lectures of Dr. L. C. Ingersoll, in the dental department of State University of Iowa.

Dr. B. S. Bynus showed his improved method of regulating with gold wire, with models of cases, before and after regulating.

Adjourned to 3 P. M.

---

### SECOND DAY.—SECOND SESSION.

Called to order at 3:30 P. M., the President in the chair.

The discussion of DENTAL HYGIENE continued.

Dr. G. F. S. Wright, Columbia, S. C., did not agree with Dr. Morgan and others on the subject of Diet; he thought the excessive feeding of infants produced an unhealthy sleep, and developed other evils. The bovine species lay down when full, not to sleep, but to chew the cud. The results of eating at certain hours depended on the individual; the heartiest meal might be taken at morning, noon or night, as each individual preferred.

Defective inspiration was one great cause of trouble; respiration was an important factor which is not sufficiently thought of. We do not inflate the lungs sufficiently.

Dr. Teague, being a very modest young man, had omitted to mention a simple contrivance of his own, an armlet by which the arms are supported while at work, giving great relief and ease. As to the light, it does not make much difference whether it comes from the north, south or west, so it falls directly on the spot where the work is to be done, and not in the eyes of the operator.

President Wardlaw (calling Dr. Catching to the chair) said it was very important to understand the use of the mouth glass in throwing a strong light on the spot where it is most needed, and also to work by the reflection in glass, not trying to see the tooth itself—in that way the operator could escape the breath of the patient, avoid the shadow of his own person, and a constrained position. It might be found a little difficult to acquire the habit, but it would be found well worth the trouble. The dentist should remember to frequently straighen up and *breathe*—they too often forget to breathe. A skylight is fine both for light and ventilation and the varying light can be readily regulated.

Dr. Freeman, Nashville, spoke of the great advantage of sitting while at work, using an ordinary counting-house stool, which can be easily moved about with the foot, or one side or the other of the patient. Heavy weight men who have not tried it cannot imagine what comfort there is in it.

Dr. W. H. H. Thackston said that he had just received a paper on Hygiene from Dr. Woodley, of Norfolk, Va., who had expected to be present, but had been prevented by a death in his family.

The paper was referred to the Publication Committee, and, on motion, the subject passed.

### OPERATIVE DENTISTRY.

Dr. Wm. Crenshaw, Atlanta, Ga., read a paper entitled "A Complete System Possible in Filling Teeth;" and Dr. Solomon read a paper sent by Dr. J. S. Knapp, of New Orleans. Dr. Solomon said he was very sorry to have to oppose the paper he had just read, but he believed in leaving the nerve if only a very little piece was left. If you take a tooth just extracted, the pulp does not fill the entire cavity; in healthy children, 17 or 18 years old, there will be from two to two and a half millimetres between the lining of the cavity and the pulp; at 30 to 40 years of age it increases to from four to four and a half millimetres. He has examined many perfectly sound teeth which had been extracted in regulating, and always finds this to be the case. If a healthy living pulp is expoused, he caps and fills immediately; if diseased and not of a healthy color, but swollen and red but no pus, he makes a very little puncture, treats with eucalyptus and iodoform for a few days, then caps and fills. If there is pus formation, he resorts to the old method, taking a sharp spoon-shaped cxcavator, and cutting out as far as possible. He then touches the pulp with a white hot instrument, so quickly that it is entirely free from pain; this treatment covers 50 per cent. of diseased pulps; no discoloration follows, and very rarely alveolar abscess. If necessary he would treat for weeks or months rather than destroy the least possible portion of live nerve. He makes a paste of calomel, oxide of zinc, and glycerine, which he pushes gently into the pulp chamber. Has seen outrageous results from the practice of Dr. Knapp in driving wood into the pulp chamber. Had one case where the patient was suffering agonies from wood driven in with a mallet; periostitis was severe, and relief not being obtained the tooth was extracted, when the wood was found extending through the end of the root; the exact length of the root cannot be measured; if the root is long, part of the nerve may be left untouched; or the wood may go beyond the apex if the root is short; he did not think it would succeed in 25 per cent. of cases.

Dr. Louis Dotterer said that, as the pulp sends out filaments into the dentine, he could not conceive how there could be any space between the pulp and the dentine, though the nerve might withdraw if killed in a vacuum. He did not approve the idea of cutting off part of the nerve; he should imagine it would be terribly painful, and would not like to have it tried on himself.

He could not agree with Dr. Crenshaw that cohesive gold was the best; soft foil was so congenial to the cervical margin, but he would add cohesive to finish; soft foil would lap well on the edges.

Dr. J. J. R. Patrick said a great deal was said about different kinds of gold, but gold was gold the world over; there is a great deal that is unnecessary and important said about the distinction between hard gold and soft gold, adhesive and cohesive, etc. If there is any difference between gold and gold, it is due to some alloy; something smeared over the surface; all that is sold as gold is not pure gold; there may be a slight addition of silver. Annealing gold makes it more tenacious, and a corner can be built out better by passing every pellet over a spirit lamp before putting it in the cavity.

Dr. McKellops wished to ask Dr. Crenshaw whether he had ever used any other mallet than the electric—Bonwill's mechanical for instance.

(Dr. Crenshaw said that he had tried them all.) Dr. McKellops said that he had one of Bonwill's first; had tried an electric mallet made in Philadelphia by Dr. Jack; had seen Dr. Jack operate with it, and had seen Dr. Bonwill use his—had been the best of friends with Marshall Webb—he always has the best money can buy, and the best of materials; had proof gold tested, from the mint of California, at $50.00 per ounce. Had wanted to make a success of the electric mallet, and liked it very well in some cases, but could not do as delicate work with it as with Bonwill's. He had seen the worst results from wedging; a front tooth lost, the whole socket being ruined by rapid process—by gradually opening with tape, in five or six days you have all the room wanted without soreness. Want of care and time will always produce disastrous results. What he had been watching lately, done by "that man from over the water," looks as if it was going to be the coming thing. It requires a little care; a man must discriminate between right and wrong, and not slobber over his work. The new method (Herbst's) offers great protection for thin walls, lapping over and protecting them well. The use of his matrix saves over half the cutting in finishing up, and saves material. It is a good thing in proper hands. All do not work alike, but would not condemn a method because it is "not my way." My friend Brown works very differently from me, but he does as pretty work as ever man did. We cannot say this is *the best* or this is *the only* way. Each must work out his own individuality. Would not like to undertake the method described by Dr. Salomon, and has never found the shrinkage described by him; finds the pulp sometimes prominent in the branches of the cusps; there is no shrinkage of pulp in young teeth, it is only in old age, and then the space is filled up by tooth bone, following the shrinkage up closely.

Dr. Morgan wished to ask Dr. Patrick why he would anneal his gold, piece by piece, a second time, when it had already been annealed in the manufacturing?

Dr. Patrick said that even if previously annealed, there were surface accumulations, and moisture that required to be drawn off before it was ready for use.

Dr. Morgan: Dr. Patrick said that many teeth were preserved, and gold fillings made with soft foil, but they were not equal to cohesive gold fillings. A few days ago I looked at fillings I made in 1848, and which are still standing without a flaw. Do you want anything better than that?

Dr. Patrick: When gold is rolled or beaten, the molecules are compressed and made smaller. They expand again to their natural form when annealed, and we use the mallet to compress and draw them together again, producing greater solidity than if we attempt to drive unannealed gold. Annealed is more compressible.

Dr. Beech was sorry to find so few disposed to advocate the virtues of non-cohesive gold. The fillings made by Dr. Morgan have stood the test of forty years! Dentistry is a conservative profession, but the man who claims that he must have a specific gold, of a specific character, to make good operations, takes a narrow view. To make the best operations it is necessary to be able to combine the best materials and the best methods. It may even be necessary to use cohesive and non-cohesive, interchangably, in the same cavity to make a perfect operation; we must use whatever is necessary to obtain the best results. In a large majority of cavities we need a combination. Do not like the terms hard and soft for gold. When you say cohesive and non-cohesive, it explains itself—one slides and one sticks. A man who confines himself to one material deprives himself of many advantages in the salvation of teeth. Root canals can be filled with wood or gutta percha, or oxychloride, or gold or tin; it makes little difference which, so that it is perfectly filled. For fifteen years I have advocated filling with lead, and have yet to find a failure. There is a chemical reason for this. A lead bullet will not cause suppuration; nature tolerates it, and this is true of a lead root filling, even if driven through the foramen. A spindle of soft lead can be driven in and so battered down, as to make a perfect root filling.

Dr. Winkler said he early made it his motto to learn to operate after the method of every good man, for each one had his own method. He said that Dr. Beech had stolen his thunder, and made his speech on that point. It was ever thought that the height of scientific skill had been attained when a tooth was filled with soft gold. All cohesive gold required an operator of exceptional skill; but the two conjoined filled every requirement, Defend the cervical walls by putting in soft gold; when within 1-16 of an inch of the edge, weld on cohesive gold and contour. Many able operators were looking in every direction for some non-conductor with which to line cavities to be built out on the surface with gold. Tin foil at the cervical margin protects from all danger of leakage, or of fracture with the mallet, but tin foil darkens the teeth. A subsequent operator seeing this dark streak might think it a renewal of decay, but on testing would find both filling and tooth perfect. Dr. Webb probably pronounced tin foil the most difficult material to fill with, simply because he had not used it enough to know the contrary. There is nothing so easy to work, but it requires peculiar manipulation.

Dr. Patrick asked Dr. Winkler to name the difference between soft foil and adhesive gold?

Dr. Winkler said that only, that one would slide on itself—the other would adhere.

Dr. Patrick said it was only necessary to anneal the gold, drive off all moisture, to make it adhere.

Dr. Winkler spoke of the different methods employed by different operators—the electric mallet, hand pressure, the lead mallet, minutely serated instruments, or polished agate or ivory points,—and yet each one may make beautiful work. What is perfect for one will not suit another at all, and each one must choose what suits his capacity best.

Dr. Morgan said he was very glad to hear the statement, or the inference from the statement made, that all gold was chemically the same; that cohesive and non-cohesive simply grows out of the relations of the molecules. Pure gold is cohesive; non-cohesive gold is not clean, not pure. Dr. Dwinelle has used only sponge gold for many years. In soft gold there is not such perfect adhesion; small particles scale. He did not agree with what had been said about lead. A tooth without pulp was dead to all intents and purposes, only the cementum being nourish ed. As to the root filling acting as an irritant, and requiring toleration, it might as well be put in a body, as far as coming in contact with living tissues is concerned. The life of the dentine was destroyed with the pulp; the cementum stands between the dead and the living, and makes no complaint.

Dr. McKellops asked what were the chances of success where a tooth is extracted and the cementum pared off, shaping the tooth to fit a socket made to receive it, as described by Dr. Younger, of California?

Dr. Morgan thought that if the root was well polished nature might tolerate it, by encysting like a bit of steel or gold in the hand. It might be tolerated, or it might be ejected like a splinter. In any case the tooth must be thoroughly divested of all septic conditions.

On motion adjourned to 9 A. M.

---

THURSDAY, July 2nd.

THIRD DAY.—MORNING SESSION.

Devoted to clinics, (in lower hall, Watkins' Institute.)

Dr. G. H. Winkler, Augusta, Ga., filled a second superior molar, buccal and crown cavity, with non-cohesive foil, using old style forcep-pluggers for condensing his gold, giving great power with ease, to the operator, and safety to the patient. Dr. Winkler also filled proximal cavities in the central incisors, the fillings scarcely showing in the front.

Dr. W. H. Richards, Knoxville, Tenn., filled a second superior molar, crown and buccal cavity, using the hand mallet, soft foil, smooth pluggers, and Justi's speculum or reflector. The rubber dam was secured in place between the teeth by beads passed on the ligature.

Dr. How, Philadelphia, exhibited the appliances for his crown and bridgework; Dr. J. R. Knapp, New Orleans, his crowns and blowpipe; Dr. J. J. R. Patrick, his apparatus for the expeditious manufacture of gold crowns; Dr. M. W. Williams, Hopkinsville, Ky., his new motive power for engines, lathes, etc.; Dr. B. H. Teague, Aiken, S. C., his depressed disks, for dressing contour fillings, and thick rubber disks to place on the ball of the thumb, used in polishing rubber or other plates; Dr. B. S. Byrnes, Memphis, Tenn., a double clamp for holding bibulous paper in place, instead of employing the rubber dam for simple operations; also a finishing burr for cutting off roots for the attachment of crowns—a burr which can be guided like a pen, and passes under the gums without wounding the tissue.

---

THIRD DAY.—3 P. M.

Called to order by the President.

Dr. F. S. Chisholm, acting secretary, read a communication from the faculty of Vanderbilt University, inviting the members of the Association to visit the grounds and buildings.

On motion of Dr. James Johnston the invitation was accepted, and a resolution of thanks ordered.

The discussion of

OPERATIVE DENTISTRY

was continued. Dr. Catching recommended the reception of a paper from Prof. Gorgas, on the subject of Dental

Education. The paper was read by title and referred to the publication committee. The subject of Operative Dentistry was passed, and

HISTOLOGY AND MICROSCOPY

called. Dr. E. S. Chisholm read the only paper, and the subject was opened for discussion.

Dr. Chisholm said he wished to make an explanation. There seemed to be a disposition to receive opinions rather than ask demonstration, but we should guard well that point, and demand demonstration, not statements; we have all heard the statements made about pus exuding around the gums; it may be pus, and it may not be.

Dr. Freeman said that he did not profess to be posted in microscopy, but he had a very nice specimen of tooth-germination, showing the papilla, colored with chloride of gold.

No further remarks being made, the subject was passed and

PATHOLOGY AND THERAPEUTICS

called.

Dr. R. B. Adair, Gainesville, Ga., was introduced by the President as an expert in the treatment of pyorrhea alveolaris, but who, not having been able to find a patient for his promised clinic, would take the floor and explain his method.

Dr. Adair said that he had not come prepared to read a paper, and still less to make a speech.

Riggs' Disease was so common in some parts of the country that it was very important to have a thorough knowledge of its etiology and pathology. At his own home it was so common that he could show half a dozen cases at any time. He congratulated Nashville on its apparent rarity. He began the experiments, of which his present very successful mode of treatment is the result some eight years ago. Riggs' treatment was entirely surgical, which is good as far as it goes, but will not restore lost tissues. The pockets still exist, and food will continue to be forced down into them, keeping up irritation. Many experiments have been made for the protection of the parts, as plates, rubber dam, sponge grafts, etc., but all fail more or less.

Dr. Adair claims to have a remedy that meets all requirements. His treatment is as follows:

A few crystals of iodine are dissolved in enough pure wood creosote to make a saturated solution, which improves with age. This is applied down in the pockets and all over the suppurating surface. It destroys all germs and stimulates to healthy action.

A glyceride of tannin is made by packing in a small wide mouthed bottle as much crystals of tannin as it will hold, and adding enough glycerine to dissolve it, making a very thick solution. Drying the parts, this is applied all over the surface, and seals up the pockets, protecting them perfectly for twenty-four hours; the saliva flowing over forms a tannate of albumen, forming a pellicle which resists friction. The application of the two remedies must be made every day, the pellicle being first pealed off, application continuing till the pockets are filled up, and the outline of the gum restored. This will take from 10 to 120 days according to the severity of the case. He has had perfect restoration where the breath was so bad that it was necessary to disinfect the office.

Dr. George Eubank asked if he would look for success in a case where the periostuim was destroyed two-thirds the length of the root?

Dr. Adair said he should feel confident of success. In reply to a question, he stated that tincture of iodine would not answer.

Dr. B. H. Catching said that he knew of Dr. Adair's great success, but that he succeeded, also, by a different method. He believed there was always caries of the margins of the process, and that it was necessary to trim that away very thoroughly first, and then apply aromatic sulphuric acid. Then (being governed by circumstances) apply the iodine, or chloride of zinc, forty grains to the ounce. For each case he purchased a sable hair brush, (*not* camel's hair,) writing the name of the patient on it, and burning it when the case is dismissed. Every part can be reached with this brush without irritation.

Dr. G. Chisholm, Columbus, Miss., succeeded very well with Dr. Adair's method, when patients could be induced to return for treatment, but they were very apt to stay away as long as comfortable. He prescribed, in addition, a mouth wash, as follows:

| | |
|---|---|
| Crystal iodine, | gr iij; |
| Tincture aconite, | ʒ iij; |
| Myrrh, | ℥ j; |
| Tannin, | grs x; |

Alcohol sufficient to make 3 ounces, and flavored with wintergreen.

Use a few drops on the brush, without water. This keeps the mouth in good order; and as more pus forms, the main point is to remove all debris and necrosed and bone. Nothing equals the tannate of glycerine for protection. The salivaflowover forms a tannate of albumen which keeps all foreign substances out.

Dr. Salomon asked if he had ever cured cases caused by uric acid? *

Dr. Adair said that he did not know whether or not it was caused by uric acid, but his treatment always succeeded. He thought the disease was sometimes local, and sometimes hereditary. He had not studied the etiology thoroughly.

Dr. Morgan said it was fundamentally important to get at the pathology. It was a fundamental principle to remove the cause. Unless this was understood, the practice was purely empirical, quackish. Those who are successful should give their views of the pathology and cause;

* **As suggested by Dr. Reese, of Galveston, in a paper read before the Louisiana Dental Association, in New Orleans, in 188[illegible].**

otherwise we are wandering in the dark. But he did not mean to say that the gentlemen who had spoken were quacks, or were practising from that standpoint.

Dr. Catching said that he was not speaking of the pathology of pyorrhea—only of Dr. Adair's successful treatment.

Dr. Crawford said that he believed the disease had its origin, primarily, in the cementum. The pericementum was the first structure to make outcry, though he was not prepared to say that the primary lesion was in the cementum. The great trouble in his mind was to differentiate between true pyorrhea, and local trouble, or the result of pure automatism. If the disease depends on constitutional or dietetic conditions, we must go beyond local or topical treatment, and build up the constitution by general treatment. Mere palliative measures, or attempted restoration of the parts, will not prevent their recurrence. If we have the destructive type, depending on constitutional causes, or hereditament, a cure is beyond the range of our abilities. If traumatic in character, and amenable to surgical treatment, nothing is better for healthy granulation and new tissues, than to wash out, dry with absorbents, and touch with pure carbolic acid. Has never had as satisfactory results from iodine as from carbolic acid.

Dr. Thackston said that he must be pardoned for referring to the pathology of half a century ago. He was the only one left to represent the doctrine then taught in the Baltimore College—taught by such astute, careful observers, as Horace Hayden, one of the fathers of American dentistry. After years of patient investigation of this disease, not known then as pyorrhea alveolaris, nor as Riggs' Disease, his idea was that it was a conjoined suppuration of the gums and alveolar process. He taught that while local, it was a local manifestation of constitutional disturbance; vitiated and impaired nutrition of the general system. The treatment must comprise and embody the building up and repair of the whole body; to keep in abeyance the ravages of that disease for which there was no radical cure, in his opinion. After patient employment of all the remedies suggested, he concluded that conjoined suppuration of the gums and alveolar process could never be cured, though it could be abated, modified, or palliated, with the co-operation of intelligent, careful patients. The success of more modern systems of treatment, dating from the teachings of Dr. Riggs, is a long ways ahead of anything done by Hayden or students of that day. He was undoubtedly entirely correct in the removal of all local causes; the tonic treatment of to-day was also more reliable and the true method. These gentlemen appear to have fallen upon a combination which is wonderfully successful, and adds greatly to our resources; all honor to them, though I still cling to the idea of combining general with local treatment, as remedial and alterative, with surgical treatment also necessary.—*Southern Dental Journal.*

---

ARTICLE II.

### MEDICAL EDUCATION FOR DENTISTS, AND DENTAL EDUCATION FOR PHYSICIANS.

READ BY B. H. CATCHING.

**[Before the Southern Dental Association.]**

